# Supplementary figures and images for: Exploring the Extracellular Regulation of the Tumor Angiogenic Interaction Network Using a Systems Biology Model
Source: Front Physiol. 2019 Jul 18;10:823. doi: 10.3389/fphys.2019.00823 (PMC6656929; doi:10.3389/fphys.2019.00823)

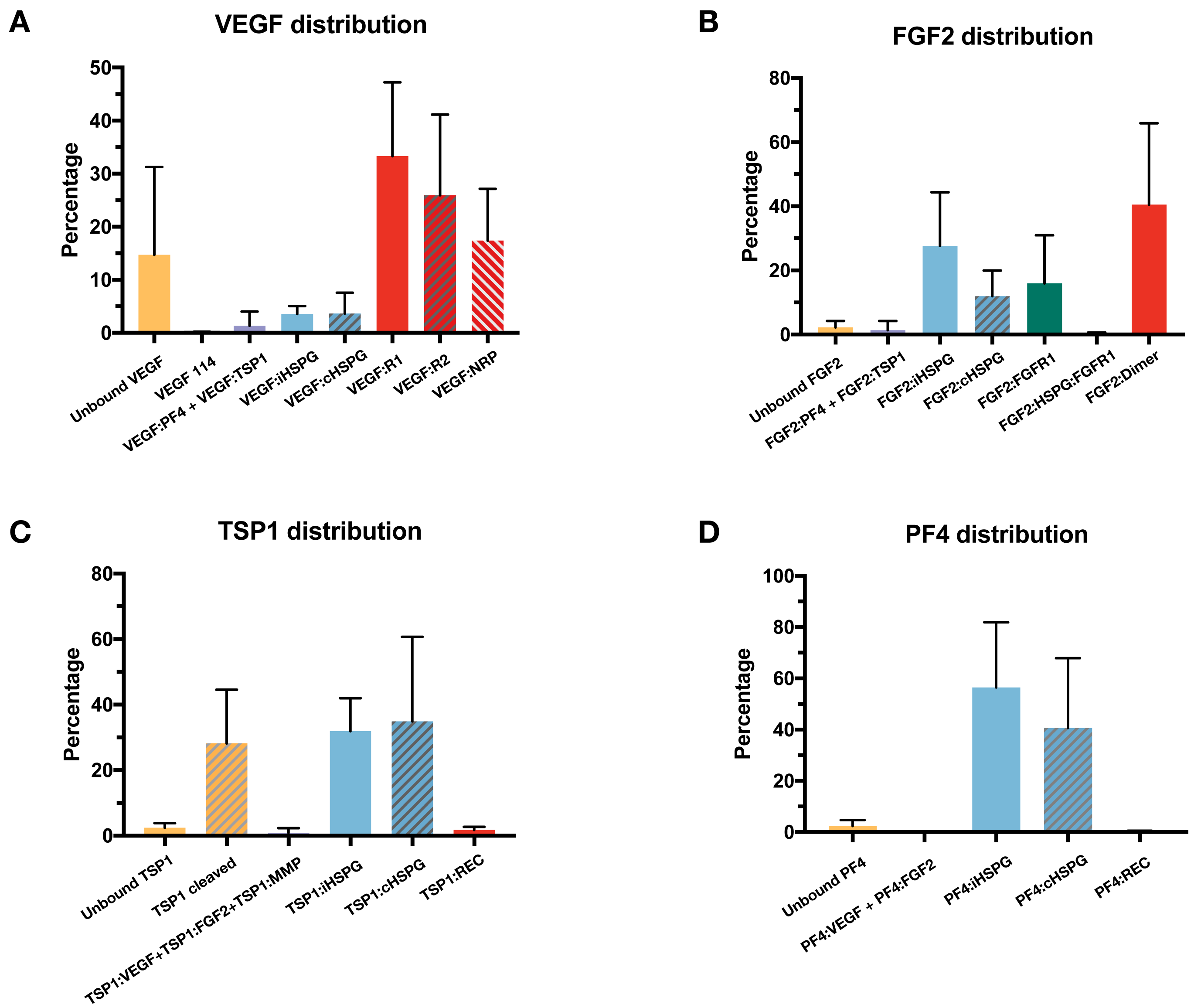

Supplement: FIGURE S1 — The variations of predicted tumor tissue distribution of VEGF (A), FGF2 (B), TSP1 (C), and PF4 (D). The secretion rates of VEGF, FGF2, TSP1, PF4, and MMPs are sampled within a range of 100-fold below and 10-fold above the baseline values. The mean value and the standard deviations of the predictions of 5000 Monte Carlo simulations are shown in plots. [file Image_1.TIF]

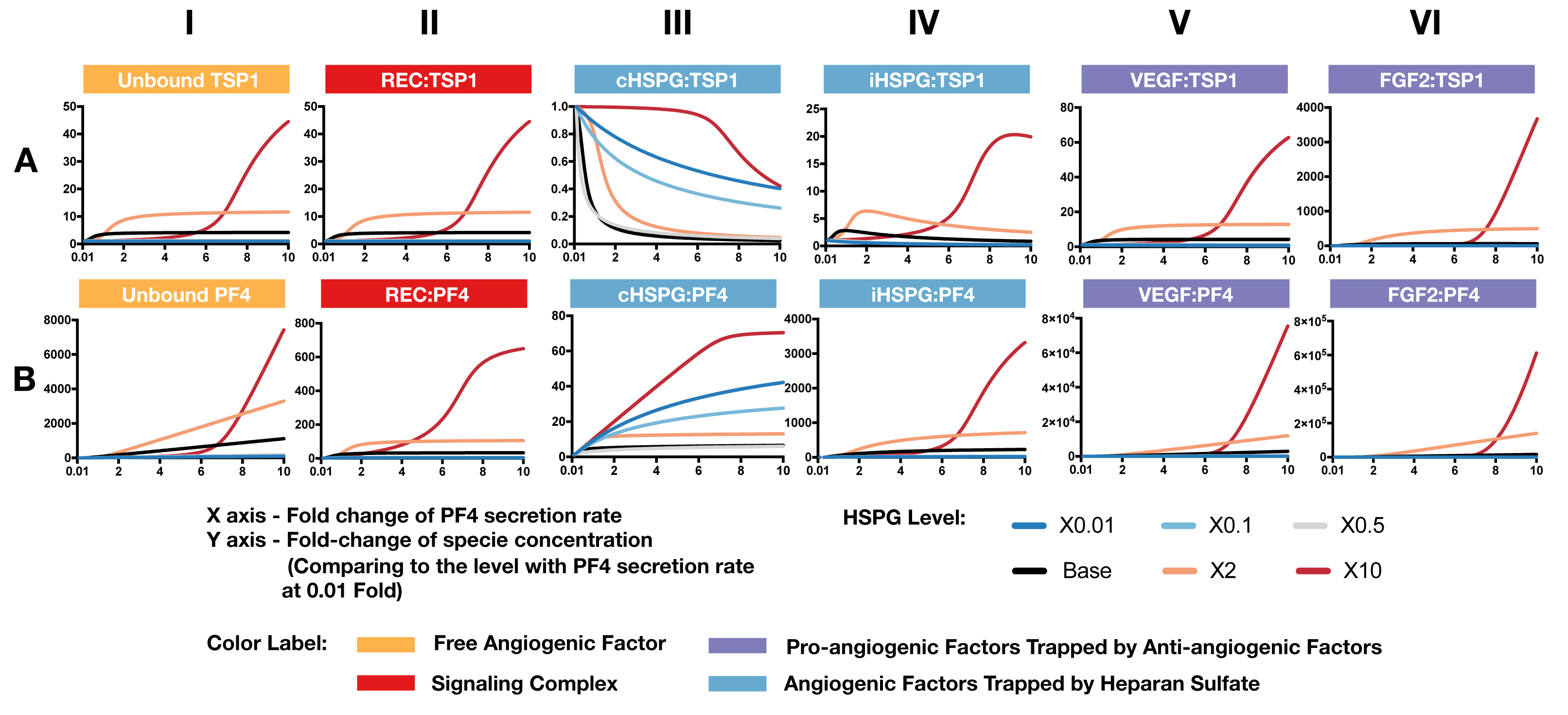

Supplement: FIGURE S2 — Effects of PF4 secretion on the angiogenic distribution. The predicted change of (A) TSP1 and (B) PF4 species with increasing PF4 secretion. [file Image_2.TIF]
